# Lethal and Sub-Lethal Effects of Insecticides on the Pink Hibiscus Mealybug, *Maconellicoccus hirsutus* (Hemiptera: Pseudococcidae)

**DOI:** 10.3390/insects10010031

**Published:** 2019-01-16

**Authors:** Fatemeh Ganjisaffar, Sharon A. Andreason, Thomas M. Perring

**Affiliations:** Department of Entomology, University of California, 900 University Ave., Riverside, CA 92521, USA; sharon.andreason@ucr.edu (S.A.A.); thomas.perring@ucr.edu (T.M.P.)

**Keywords:** toxicity, feeding disruption, spray bioassay, date palm, synthetic insecticides

## Abstract

The pink hibiscus mealybug, *Maconellicoccus hirsutus* (Green) (Hemiptera: Pseudococcidae) is a pest of many plants, and a new problem on dates in California. The effects of seven insecticides and water on different life stages of this mealybug were studied to identify the best material for control. Water did not have any significant effect on mealybugs, but the insecticide treatments significantly affected all life stages tested. The egg hatch rate ranged from 28.5% to 17.2% for spirotetramat, bifenthrin, flupyradifurone, fenpropathrin, and buprofezin treatments, and was lower for sulfoxaflor (2.8%) and acetamiprid (0.1%). Despite high survival of neonate crawlers in the non-treated control and water treatments, 53.1% and 34.6% survived in the spirotetramat and buprofezin treatments, respectively; survival was zero in the other treatments. Spirotetramat and buprofezin caused very low mortality of nymphs in the first day post-treatment, but mortality significantly increased over time and reached 42.8% and 50.6% by day 6, respectively. The other treatments were highly toxic to the nymphs (79.4–99.4% on day 6). Insecticides also had a significant effect on the feeding ability of nymphs. By day 6 after treatment, 73.9% to 100% of nymphs treated with different insecticides stopped feeding although they were still alive. Insecticides showed no effect on the mortality of adult females, but the percentages of ovipositing females were significantly reduced (51.1% to 10.6%) in all insecticide treatments, except buprofezin, which was not statistically different from water and the non-treated control. In the process of our studies, we identified abnormalities in the appearance of eggs from females treated with various insecticides, and these aberrant eggs are described.

## 1. Introduction

The pink hibiscus mealybug (PHM), *Maconellicoccus hirsutus* (Green) (Hemiptera: Pseudococcidae), is thought to be native to southern Asia as it first was collected and described in India in 1908 [1,2]. The first invasion of the pest was reported from Egypt in 1925 [3]. Since then, it has spread to other tropical, subtropical, and temperate regions of the world including the rest of Africa [4,5,6,7], the Middle East [8,9], Australia [10], the Caribbean Islands [11,12], and the Americas [13,14,15,16,17,18]. In the United States and related territories, PHM first was detected in Hawaii in 1983 [19] and has been established in the Mariana Islands [20], Puerto Rico [13,21], California [22], Florida [23] and the Virgin Islands [24]. In addition, infestations have been reported from Alabama and Oklahoma (2005), Louisiana (2006), New York and Texas (2007), Georgia (2008), North and South Carolina (2009), and Tennessee (2014) according to the National Agricultural Pest Information System (NAPIS) [25].

Pink hibiscus mealybug is a polyphagous sap-sucking insect that feeds on a wide range of host plants in 76 plant families and over 200 genera [26,27,28]. Economic losses occur in forest trees and ornamentals, field crops, vegetables, and fruit trees such as citrus, grapes, and avocados [13,23,29,30,31]. Feeding damage by PHM results in deformed fruits, leaves, and shoots, stunted plant growth, and eventual plant death [13,32]. The pink hibiscus mealybug is considered a serious pest in the United States, due to its extremely broad range of economically important hosts [33]. Estimated economic losses due to its damage and control have been predicted to reach $700 million in the United States [34] and $5 billion globally [35].

In California, the first introduction of PHM was into the Imperial Valley (Imperial Co., CA, USA) in the fall of 1999. A biological control program by the California Department of Food and Agriculture (CDFA) was established, and two exotic encyrtid parasitoid wasps, *Anagyrus kamali* Moursi and *Gyranusoidea indica* Shaffee were released from 2000–2004. This program resulted in the establishment of the parasitoids and reduction of PHM to nearly undetectable levels [36]. Pink hibiscus mealybug was detected further north in the Coachella Valley (Riverside Co., CA, USA) in 2009 and slowly spread northward until 2014 when it was found to be widespread on landscape plants in the urban areas from Indian Wells to Palm Springs. To address this problem, we initiated a biological control program in which we released *Anagyrus callidus* Triapitsyn, Andreason & Perring (originally thought to be *A. kamali* but described as a new species [37]) and *G. indica*.

**Figure 1:**
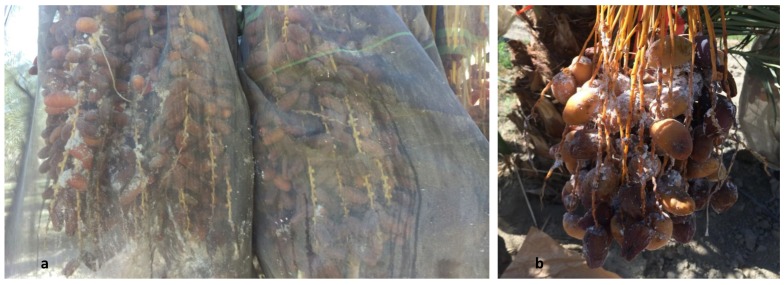
(**a**) Date bunches (variety Medjool) covered with polyester mesh bags and infested with pink hibiscus mealybug. The covering can limit biological control using parasitoid wasps. (**b**) A date bunch (variety Medjool) with heavy infestations of pink hibiscus mealybug.

In addition to infestations on ornamentals, PHM has been identified in fruit bunches of date palms growing in the Coachella Valley. Although classical biological control has proven to be effective in urban regions of the Imperial and Coachella Valleys, the use of parasitoids in dates is limited. A cultural practice used to prevent insect infestation (especially carob moth, *Ectomyelois ceratoniae* (Zeller)) and reduce rain damage which can lead to fruit rotting [38], is covering the fruit bunches with polyester mesh bags [39]. These bunch covers also prevent access of natural enemies to pests in the bunches and mealybug populations can build up rapidly in dates that become infested before the bunches are covered (Figure 1). Therefore, the timing of control is critical to ensure bunches are pest free before covering. In addition, it is important to provide date producers with a range of control options from which a holistic management program can be developed. The goal of this study was to evaluate the toxicity of seven insecticides with different modes of action to identify the best candidates for PHM control.

## 2. Materials and Methods

### 2.1. Insect Rearing

Terminal branches of the carob tree (*Ceratonia siliqua* L.) infested with PHM were collected from the Coachella Valley in the fall of 2015 and transported to the University of California, Riverside (UCR) Insectary and Quarantine facility under the appropriate permit. Colonies were started and have been maintained on squash fruit (*Cucurbita moschata* Duchesne ex Poir., varieties Black Futsu and Shishigatani) grown at the Agricultural Operations at UCR. Harvested squash were washed to remove dirt and any arthropods, decontaminated in a 5% bleach solution for 5 min, then transferred to a container of water for a few minutes before a final rinse in distilled water to remove bleach residues. The squash fruits were infested with first instar nymphs (crawlers) which were attracted to a pinpoint light within a dark rearing cabinet. The crawlers were sprinkled onto clean squash using a flour sifter every day. Mealybugs infested the squash and were kept in a dark cabinet where they develop into adults. Males and females mated, and their progeny provided a consistent supply of crawlers to re-infest new squash. The PHM colony was maintained at 26±2 °C, 50±10% RH, and 0:24 L:D photoperiod.

### 2.2. Experimental Arenas

For experimental arenas, we needed a substrate that could last for about 10 days (duration of the bioassay) allowing us to evaluate the effect of different treatments for 6 days after treatment. Also, it was important that the substrate could support the development of PHM. Date fruits and squash fruits and leaves of some common host plants, Chinese hibiscus (*Hibiscus rosa-sinensis* L.), squash, and cotton (*Gossypium hirsutum* L.), were tested for their suitability in experimental arenas. After these preliminary tests, cotton leaves were selected based on the criteria mentioned above. The cotton plants were grown in a greenhouse until they reached a height of 50–60 cm, after which they were moved to the laboratory to make experimental arenas. Arenas consisted of 6×1.5 cm Petri dishes, containing a substrate of 0.5% agar (MP Biomedicals, Santa Ana, CA, USA). A cotton leaf disc (4 cm in diameter) was placed with the abaxial side facing up on top of the agar. The 0.5% agar concentration provided a semi-solid substrate that kept leaves from desiccating. A 1-cm hole was made in the lid of the Petri dish and covered with fine mesh for ventilation while not allowing escape of mealybugs.

### 2.3. Insecticides Evaluated and Treatment Methodology

The materials selected for this bioassay were those used against mealybugs or similar pests in other crops, and they represented a wide range of modes of actions. Insecticides tested were flupyradifurone, spirotetramat, sulfoxaflor, buprofezin, acetamiprid, bifenthrin, and fenpropathrin (Table 1). For each insecticide, the maximum label rate for mealybugs or similar pests on another agricultural commodity was identified, and the concentration of this rate in 935 L/ha of water was determined (Table 1). Each material was mixed with distilled water using this concentration, placed in the reservoir of a deluxe airbrush (Central Pneumatic #69492, Camarillo, CA, USA) set at 5 psi and held perpendicular at 15 cm away from the experimental arenas inhabited by mealybugs. The effect of distilled water also was evaluated, and there was a non-treated control.

### 2.4. Bioassays

All treatments were evaluated at the same time. After spraying the arenas, the leaves were allowed to dry for about 45 to 60 min at room temperature, and then the lids were placed on the arenas. All experimental arenas then were maintained in a growth chamber at 24±2 °C, 50±10% RH, and 0:24 L:D photoperiod during the evaluation period.

#### 2.4.1. Eggs

Gravid females were brushed off the squash in the stock colony; these squash fruits had been infested with crawlers 28 days earlier. Five Petri dishes (10×1.5 cm) were created with cotton leaves as mentioned previously, and 30 females were transferred to each dish. After 3 days, the ovisacs (waxy filamentous secretions covering eggs) were gently detached from the females using a fine brush, and they were moved individually to experimental arenas. The ovisac in each arena then was sprayed or left as a control. Each treatment was replicated 12 times (twelve arenas per treatment). Following treatments, the ovisacs were pulled apart and the total number of eggs in each ovisac was counted. The eggs were checked daily for the next 12 days. At this time, the numbers of hatched/unhatched eggs and dead/alive crawlers were recorded. A second study was conducted on eggs in which the ovisacs were not pulled apart, allowing the assessment of the treatment to intact ovisacs. For this study, ten ovisacs were placed in each arena and sprayed, after which the effects on egg hatchability and crawler survival were determined. Each treatment was replicated 10 times in this study.

#### 2.4.2. Nymphs

Second-instar nymphs were brushed off a 14-day-old infested squash from the stock colony. Twenty nymphs were transferred to each arena, and allowed to settle for 24 h, after which 15 successfully settled individuals were kept in each arena. Each treatment was replicated 12 times. After treatments, mortality was recorded at 24 h intervals for 6 days. Mealybugs were considered dead if they failed to move their legs after gentle probing with a fine brush. In addition, the number of nymphs that were still alive but stopped feeding (probed mealybugs were unable to grip the leaf and their stylet was not inside the leaf) was recorded daily.

#### 2.4.3. Adult Females

Adult females were brushed off a 28-day-old infested squash from the stock colony. Similar to the nymph bioassay, twenty adult females were transferred to each arena from which 15 settled individuals were kept after 24 h. There were 12 replicates for each treatment. Mortality was recorded at 24 h intervals for 6 days. Mealybugs were considered dead if they failed to move legs after gentle probing with a fine brush. In addition to the mortality assessment, the effect of insecticide treatments on oviposition was determined by recording the proportion of ovipositing females in each arena at day 6 after application.

### 2.5. Statistical Analysis

All data analyses were performed using R version 3.5.1 for windows [40]. A generalized linear model (GLM) with a binary response (dead/alive) and a binomial distribution (glm function) was applied to test the effects of different treatments on mortality of the nymphs and adult females. The GLM was followed by Tukey’s test (glht function) to compare the mean mortality rates among different treatments (*p* < 0.01). For the number of nymphs that stopped feeding, the data were analyzed using Fisher’s Exact test followed by pairwise comparisons between treatments (cldlist function) (*p* < 0.01). The variances in the percentage of hatched eggs and the percentage of ovipositing females were heterogeneous (*p* < 0.05), therefore they were subjected to one-way ANOVA followed by Games-Howell posthoc test (*p* < 0.05).

## 3. Results

Insecticide treatments had a significant effect on the average hatch rate of the mealybug eggs (*F*${}_{8,\;98}$ = 76.58, *p* < 0.001) (Table 2). The highest hatch rates were observed in the non-treated control (85.7%) and water (78.4%) treatments, which were not statistically different from each other. The hatch rate declined significantly in the insecticide treatments, and was not significantly different between spirotetramat, bifenthrin, flupyradifurone, fenpropathrin, and buprofezin treatments (ranging from 28.5% to 17.2%). Sulfoxaflor and acetamiprid treatments showed significantly lower hatch rates (2.8% and 0.1%, respectively). A high percentage of crawlers survived after emergence in the non-treated control and water treatments (93.2% and 82.9%, respectively). Treatments with the two growth regulators, spirotetramat and buprofezin, resulted to 53.1% and 34.6% crawler survival, respectively. However, the crawler survival in other treatments was negligible or zero (Table 2). The statistical comparison of the survival rates among the treatments was not possible because only one of the eggs in one of the replicates in the acetamiprid treatment hatched, and there was an insufficient number of replicates to perform the analysis.

**Figure 2:**
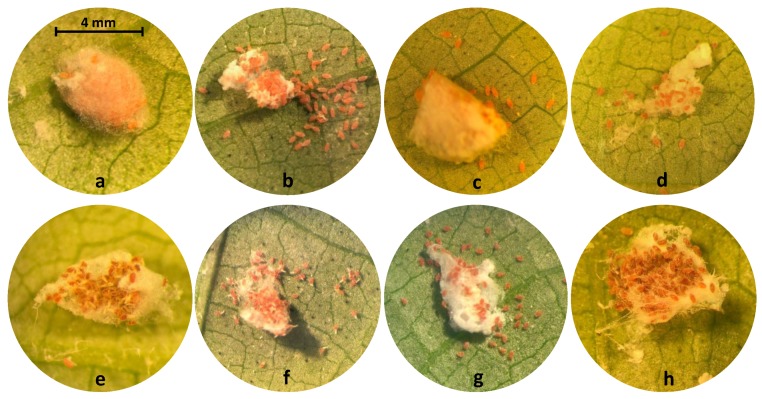
The effects of different treatments on the eggs of *Maconellicoccus hirsutus* in undisturbed ovisacs and survival of the emerged crawlers after egg hatching: (**a**) An ovisac before treatment (**b**) Non-treated control and water: High egg hatchability and survival of crawlers (**c**) Spirotetramat: Low hatch rate of eggs but medium survival of crawlers (**d**) Buprofezin: Very low hatch rate of eggs and low survival of crawlers (**e**) Bifenthrin: Low egg hatch and zero survival of crawlers. Crawlers died within the ovisac. (**f**) Fenpropathrin: Crawlers were able to leave the ovisac but died shortly close to it with the egg chorion still attached to the nymph. (**g**) Flupyradifurone: Most of the crawlers could emerge from the egg choroin and leave the ovisac but died shortly close to it. Sulfoxaflor resulted in the same crawler mortality but a much lower number of eggs hatched. (**h**) Acetamiprid: The hatch rate was almost zero.

Observations of treated ovisacs provided additional information about the effects of insecticides on the emerged crawlers (Figure 2). In the non-treated control and water treatments, a high percentage of the eggs hatched, and the crawlers survived normally. In treatments with the growth regulators, spirotetramat and buprofezin, a much lower number of eggs hatched and some of the crawlers survived (Figure 2c,d, respectively). For ovisacs treated with bifenthrin, the eggs hatched but the emerged crawlers were not able to leave the ovisac and died within it (Figure 2e). Crawlers in the fenpropathrin treatment were able to leave the ovisac but died close to it with the chorion still attached to the nymph (Figure 2f). In the flupyradifurone treatment, most of the crawlers could emerge from the egg chorion and leave the ovisac but died close to it (Figure 2g). Sulfoxaflor resulted in the same crawler mortality but a much lower number of eggs hatched. The hatch rate was almost zero in the ovisacs treated with acetamiprid (Figure 2h).

Mortality of PHM nymphs was significantly affected by different treatments at all time intervals (*p* < 0.01) (Table 3). Mortality with water was not significantly different from the non-treated control from day 1 to day 6. Spirotetramat caused low mortality in the first two days (0.6% and 8.9%) which were not statistically different from the non-treated control and water. However, the mortality by this growth regulator increased over time and reached 42.8% by day 6 (71-fold increase from day 1 to day 6). The other growth regulator, buprofezin, showed a significant difference from the water and non-treated control beginning at day 4, and caused 50.6% mortality at day 6 (13-fold increase from day 1 to day 6). Mortality with sulfoxaflor increased from 8.9% on day 1 to 79.4% on day 6 though it was not significantly different from non-treated control and water in the first day. Acetamiprid, flupyradifurone, fenpropathrin, and bifenthrin were the most toxic insecticides to the mealybug nymphs as indicated by the highest mortality rates from day 1 (27.8–60.0%) to day 6 (89.4–99.4%) (Table 3).

In addition to the acute mortality effects on nymphs, the tested insecticides had a significant impact on the feeding ability (*p* < 0.01) (Table 4). One day after spray treatments, more than 97.3% of the nymphs treated with sulfoxaflor, acetamiprid, flupyradifurone, fenpropathrin, and bifenthrin stopped feeding although they were still alive. This rate reached 100% by the second day. For spirotetramat and buprofezin, the percentages of nymphs that stopped feeding increased from 8.3% on day 1 to 75.6% and 73.9%, respectively, on day 6. There were no significant differences between these growth regulators and the other insecticides on days 5 and 6 (*p* < 0.01) (Table 4).

**Figure 3:**
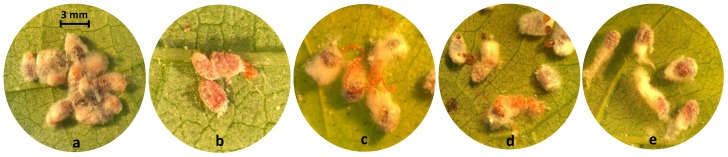
The side effects of different treatments on adult females of *Maconellicoccus hirsutus* and their oviposition behavior: (**a**) Normal oviposition and production of ovisacs in non-treated females and those treated with water and buprofezin. (**b**) Females treated with spirotetramat were not able to produce ovisacs and laid fewer scattered eggs. (**c**) Females treated with sulfoxaflor and flupyradifurone produced the wax needed for ovisacs but laid long curly strands of eggs not covered by the wax (**d**) Acetamiprid treated females produced little wax that did not cover the few eggs laid. They also made dark sticky secretions. (**e**) Females treated with fenpropathrin and bifenthrin produced waxy filamentous secretions of the ovisacs but only a few laid eggs.

**Figure 4:**
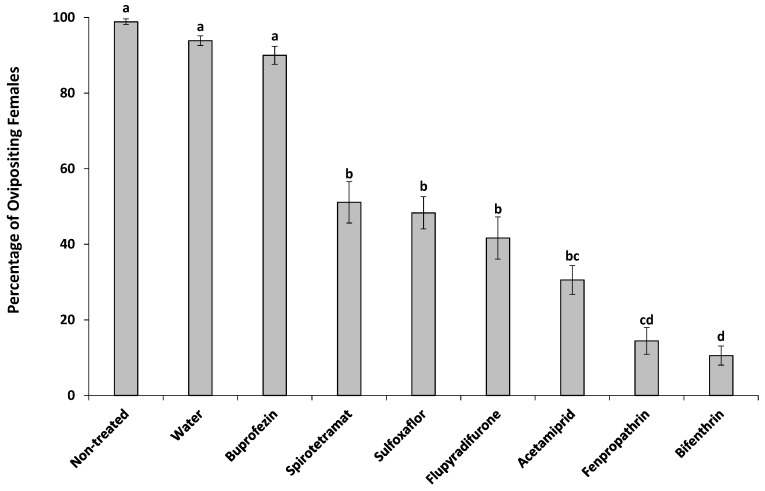
The relationship between different treatments and the percentage of adult females of *Maconellicoccus hirsutus* with successful oviposition (ANOVA summary: *F*${}_{8,\;98}$ = 76.58, *p* < 0.001). Bars with the same letter are not significantly different (Games-Howell Test, *p* < 0.05).

All tested insecticides showed zero or little effect on the mortality of adult females (<7% mortality on day 6), and there was no significant difference among the treatments (*p* < 0.01) (Table 3). Females in all treatments (except buprofezin and spirotetramat) were unable to grip the leaf and stopped feeding. However, they were still capable of ovipositing although their oviposition was compromised by the insecticide treatments (Figure 3). The symptoms of insecticide treatments ranged from no ovisac production (spirotetramat), to long curly strands of eggs (sulfoxaflor and flupyradifurone), to dark sticky secretions with very little wax (acetamiprid), to wax production with very few eggs laid (fenpropathrin and bifenthrin). Also, treatments had a significant effect on the percentage of ovipositing females in each arena (*F*${}_{8,\;98}$ = 85.86, *p* < 0.001) (Figure 4). The percentages of ovipositing females were 93.9% and 90.0% in water and buprofezin treatments, respectively, which were not significantly different from non-treated control (98.9%). Spirotetramat, sulfoxaflor, flupyradifurone, and acetamiprid had lower rates of ovipositing females ranging from 30.6% to 51.1% with no significant difference among these treatments. The lowest rate of ovipositing females was observed with bifenthrin (10.6%) which was not statistically different from fenpropathrin (14.4%) (Figure 4).

## 4. Discussion

The efficacy of a broad selection of insecticides including carbamates [41,42], organophosphates [43,44,45], neonicotinoids [41,42,44,46], pyrethroids [41,42,43], and insect growth regulators [45,47] has been evaluated on pink hibiscus mealybug in the field and laboratory bioassays. However, there is no material registered for pink hibiscus mealybug suppression on dates in California, and the main objective of this study was to identify the best chemical for that purpose. Our results indicated that all of the tested insecticides were effective on this mealybug, and their effects were enhanced with an increase in exposure time.

The two pyrethroids tested, bifenthrin and fenpropathrin, effectively controlled all PHM life stages. Of the nicotinic acetylcholine receptor (nAChR) agonists tested, acetamiprid, as its label suggests, had a strong ovicidal effect and none of the treated eggs hatched. Based on our observations, the embryo developed and the dark eye spots were visible through the semitransparent chorion, but the embryo was not able to break out of the egg. This neonicotinoid also provided a high level of nymph mortality and significantly affected reproduction of the treated females. The second nAChR agonist, flupyradifurone, also resulted in high mortality of eggs, emerged crawlers, and second-instar nymphs, and it had a significant effect on the oviposition of adults. The majority of the eggs laid by the treated females were not viable, and the crawlers in those that hatched died shortly after emergence. The third tested nAChR modulator, sulfoxaflor, was slow-acting initially but provided a moderate level of nymphal control from day 4 to 6. The lipid biosynthesis inhibitor, spirotetramat, had more activity on eggs and immature life stages of the mealybug. In addition, reproduction of adult females was reduced, and fewer eggs were laid by treated females. However, the oviposited eggs were viable and hatched. Buprofezin, the other growth regulator, did not have any effect on adult mealybugs as expected, but it was effective against the immature stages. Most of the nymphs died during molting, typical of a chitin biosynthesis inhibitor. It also adversely affected the egg hatchability of mealybugs. According to the chemical label, evidence of activity may be slower than a typical contact insecticide as treated susceptible pests may remain alive on the plant for 3–7 days. However, they stop feeding eventually leading to mortality. This is in agreement with our result that shows 50.6% mortality of nymphs at day 6 when 73.9% of the nymphs had already stopped feeding. A similar trend was obtained with the spirotetramat treatment.

The application of non-selective insecticides can result in a decrease of natural enemies, thereby bringing serious consequences in the pest population dynamics [48]. Therefore, before incorporating a chemical into the Integrated Pest Management (IPM) program of a pest, its compatibility with biological control agents that are either resident or are planned to be released should be assessed. For PHM in dates, it is essential to consider the effects of candidate chemicals on the two important parasitoids of the pink hibiscus mealybug in the Coachella Valley, *A. callidus* and *G. indica*. In addition, it is important to consider the impact on *Galendromus flumenis* (Chant), which is a key predator for another major pest of dates, the Banks grass mite, *Oligonychus pratensis* (Banks) [49,50]. Several studies have shown that bifenthrin and fenpropathrin are extremely toxic to beneficial arthropods, including the parasitoid wasps and the predatory mites [48,51,52,53,54,55]; therefore, the use of these pyrethroids should be carefully considered. Acetamiprid has been reported to have negative effects on several natural enemies [56,57,58,59,60,61,62] while being harmless to others [63,64,65]. Thus, the use of this material in the IPM program in dates should be evaluated further. Compared to most neonicotinoids, flupyradifurone and sulfoxaflor have a safer profile for beneficial arthropods, allowing control of pests with lower impact on natural enemies [66,67]. Spirotetramat is safe to *Anagyrus* sp. near *pseudococci* sensu Triapitsyn et al. (recently renamed *Anagyrus vladimiri* (Triapitsyn) [37]) as it did not show any adverse effect on the reproduction of this parasitoid species [68]. It also has been shown to be harmless to *Neoseiulus californicus* (McGregor) and *Phytoseiulus persimilis* Athias-Henriot ([63]) but had severe effects on the reproduction of *Galendromus occidentalis* (Nesbitt) [65] and it adversely affected all growth stages and fecundity of *Neoseiulus fallacis* (Garman) [69]. Furthermore, spirotetramat is known to have short residual activity, thus it may be suitable for inclusion in an IPM program on dates where we may wish to release parasitoids following insecticide treatments. Buprofezin also is safe to beneficial insects and mites. It did not have any negative effect on *Anagyrus* sp. near *pseudococci* (recently renamed *A. vladimiri*) when used for controlling citrus red mite, *Panonychus citri* McGregor in citrus orchards [70].

Knowledge of the differences in susceptibilities of PHM life stages to various insecticides can inform the choice of insecticides and determine the most accurate timing for applications. Based on the results obtained in this study, excellent control of PHM should be achieved with bifenthrin, fenpropathrin, flupyradifurone, acetamiprid, and sulfoxaflor. These materials caused nominal survival in nymphs emerging from treated ovisacs and rapid declines in nymphal feeding leading to high nymphal mortality. Applications should be made in the early season when eggs are just hatching, and crawlers are present. Later in the season, if more control is needed, applications of acetamiprid, fenpropathrin or bifenthrin may be helpful to reduce the number of later instar nymphs and suppress the reproduction of females. We suggest rotating materials with different modes of action and spectrums of activity which can delay the development of resistance.

## Figures and Tables

**Table 1:** Insecticides evaluated against pink hibiscus mealybug, *Maconellicoccus hirsutus*.

Common Name	Trade Name	Chemical Class	Mode of Action ${}^{a}$	Max. Label Rate ${}^{b}$ mL or g${}^{*}$ AI ${\mathrm{ha}}^{-1}$	Bioassay Rate μL or g${}^{*}$ AI mL${}^{-1}$ DI Water
Spirotetramat	Movento 240 SC ${}^{c}$	Tetramic acid	23	731	0.78
Buprofezin	Applaud DF ${}^{d}$	Buprofezin	16	1680 ${}^{*}$	0.00180 ${}^{*}$
Acetamiprid	Assail 70 WP ${}^{e}$	Neonicotinoid	4A	237 ${}^{*}$	0.00025 ${}^{*}$
Sulfoxaflor	Closer SC ${}^{d}$	Sulfoximine	4C	300	0.32
Flupyradifurone	Sivanto Prime ${}^{c}$	Butenolide	4D	1023	1.09
Bifenthrin	Brigade 2 EC ${}^{f}$	Pyrethroid	3	937	1.00
Fenpropathrin	Danitol 2.4 EC ${}^{g}$	Pyrethroid	3	1535	1.64

${}^{a}$ Mode of action classification was taken from Insecticide Resistance Action Committee (IRAC) Version 8.3. ${}^{b}$ Concentrations were based on an application rate of 935 liters/hectare, or 100 US gallons/acre. The asterisk symbol (*) refers to the solid insecticide formulations for which the maximum label rate and the bioassay rate were determined in g AI ha${}^{-1}$. ${}^{c}$ Bayer CropScience, Research Triangle Park, NC, USA. ${}^{d}$ Dow Agrosciences, Indianapolis, IN, USA. ${}^{e}$ United Phosphorus, Inc., King of Prussia, PA, USA. ${}^{f}$ FMC Corporation, Philadelphia, PA, USA. ${}^{g}$ Valent U.S.A LLC, Walnut Creek, CA, USA.

**Table 2:** Effects of different treatments on the mean hatch rate ± SE of *Maconellicoccus hirsutus* eggs and the mean survival rate ± SE of the emerged crawlers.

Treatment	% Hatched Eggs in Each Ovisac	% Survived Crawlers after Emergence
Non-treated	85.7±3.0 a	93.2±1.6 (12)
Water	78.4±5.2 a	82.9±3.3 (12)
Spirotetramat	28.5±4.1 b	53.1±9.4 (12)
Bifenthrin	23.4±4.1 b	0.6±0.6 (12)
Flupyradifurone	21.4±4.5 b	0.3±0.3 (12)
Fenpropathrin	20.5±2.3 b	0.0 (12)
Buprofezin	17.2±3.6 b	34.6±7.8 (12)
Sulfoxaflor	2.8±1.0 c	0.0 (7)
Acetamiprid	0.1±0.1 c	0.0 (1)

ANOVA table summary: *F*${}_{8,\;98}$ = 76.58, *p* < 0.001. Values within the same column followed by the same letter are not significantly different, Games-Howell Test (p<0.05). Number of replicates have been given in parentheses. Due to zero hatch rate in acetamiprid-treated eggs, the statistical comparison of the survival rates was not possible.

**Table 3:** Effects of different treatments on the mean mortality rate ± SE of *Maconellicoccus hirsutus* nymphs and adult females for six days post-treatment.

Stage Tested	Treatment	% Mortality					
		1 d	2 d	3 d	4 d	5 d	6 d
**Nymphs**	Non-treated	0.6±0.6 a	1.7±1.2 a	4.4±1.9 a	8.3±1.9 a	10.0±1.7 a	12.2±2.0 a
	Water	1.1±0.8 a	2.8±1.0 a	7.8±1.6 a,b	10.6±1.9 a	11.7±2.0 a	15.0±2.2 a
	Spirotetramat	0.6±0.6 a	8.9±2.5 a,b	18.9±2.1 b	30.6±1.7 b	39.4±2.1 b	42.8±1.9 b
	Buprofezin	3.9±1.5 a	10.0±2.2 a,b	13.3±2.7 a,b	26.1±2.2 b	38.3±2.0 b	50.6±2.9 b
	Sulfoxaflor	8.9±1.9 a	22.8±2.7 b	38.9±3.7 c	61.1±4.4 c	72.2±4.2 c	79.4±2.9 c
	Acetamiprid	27.8±3.9 b	50.6±2.7 c	69.4±2.8 d	78.5±3.5 d	85.6±2.3 c,d	89.4±2.2 c,d
	Flupyradifurone	60.0±2.5 c	73.3±1.8 d	87.2±2.2 e	93.9±2.2 e	96.1±1.9 d	97.8±1.3 d
	Fenpropathrin	45.0±3.1 b,c	60.0±4.6 c,d	78.3±4.4 d,e	90.0±2.7 d,e	97.2±1.3 d	98.9±0.7 d
	Bifenthrin	47.2±5.1 c	70.0±5.9 d	83.3±5.0 d,e	92.8±3.1 d,e	98.9±0.7 d	99.4±0.6 d
**Adults (♀)**	Non-treated	0.0 a	0.0 a	0.0 a	0.0 a	0.0 a	0.0 a
	Water	0.0 a	0.0 a	0.0 a	0.0 a	0.0 a	0.0 a
	Spirotetramat	0.0 a	0.0 a	0.0 a	0.0 a	0.0 a	0.6±0.6 a
	Buprofezin	0.0 a	0.0 a	0.0 a	0.6±0.6 a	0.6±0.6 a	1.1±0.7 a
	Sulfoxaflor	0.0 a	0.0 a	0.0 a	0.6±0.6 a	1.7±0.9 a	1.7±0.9 a
	Acetamiprid	0.0 a	0.6±0.6 a	0.6±0.6 a	1.1±0.7 a	2.2±1.3 a	2.2±1.3 a
	Flupyradifurone	0.0 a	0.0 a	0.6±0.6 a	1.7±0.9 a	4.4±1.3 a	6.1±1.9 a
	Fenpropathrin	1.1±0.7 a	1.1±0.7 a	1.1±0.7 a	1.7±0.9 a	5.0±1.5 a	6.7±2.0 a
	Bifenthrin	0.6±0.6 a	0.6±0.6 a	1.1±0.7 a	1.7±0.9 a	3.3±1.5 a	3.3±1.5 a

Values within the same column followed by the same letter are not significantly different, Tukey’s test (p<0.01).

**Table 4:** Effects of different treatments on the mean ± SE percentage of *Maconellicoccus hirsutus* nymphs that stopped feeding for six days post-treatment.

Treatment	% Nymphs That Stopped Feeding					
	1 d	2 d	3 d	4 d	5 d	6 d
Non-treated	1.1±0.7 a	2.2±1.3 a	5.0±1.9 a	8.9±1.7 a	10.6±1.5 a	12.8±1.7 a
Water	1.7±0.9 a	3.3±1.0 a	8.3±1.7 a	11.1±2.1 a	12.2±2.1 a	15.6±2.2 a
Spirotetramat	8.3±0.9 a	25.0±1.9 a	42.2±3.1 a	50.0±3.2 a	64.4±2.2 ab	75.6±2.2 b
Buprofezin	8.3±0.9 a	19.4±2.1 a	41.1±3.6 a	48.9±3.0 a	56.1±3.7 ab	73.9±3.5 b
Sulfoxaflor	98.3±0.9 b	100.0 b	100.0 b	100.0 b	100.0 b	100.0 b
Acetamiprid	97.3±1.3 b	100.0 b	100.0 b	100.0 b	100.0 b	100.0 b
Flupyradifurone	100.0 b	100.0 b	100.0 b	100.0 b	100.0 b	100.0 b
Fenpropathrin	100.0 b	100.0 b	100.0 b	100.0 b	100.0 b	100.0 b
Bifenthrin	100.0 b	100.0 b	100.0 b	100.0 b	100.0 b	100.0 b

Values within the same column followed by the same letter are not significantly different, Fisher’s Exact test (p<0.01).
